# Supplementary material for: Resistant Starch Type 2 from Wheat Reduces Postprandial Glycemic Response with Concurrent Alterations in Gut Microbiota Composition
Source: Nutrients. 2021 Feb 17;13(2):645. doi: 10.3390/nu13020645 (PMC7922998; doi:10.3390/nu13020645)
Supplement: Supplementary file 1 [file nutrients-13-00645-s001.pdf]

Supplementary Material

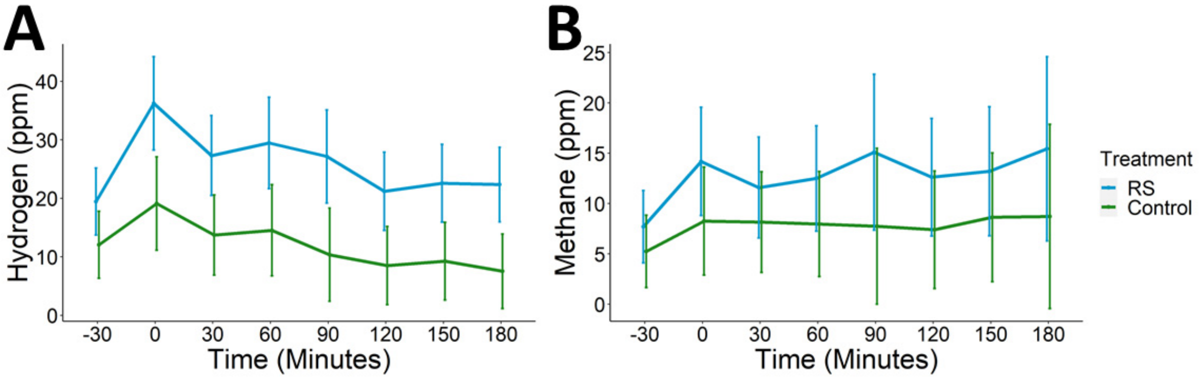

Figure S1. Postprandial curves. Postprandial curves shown for A) hydrogen and B) methane. Values shown are mean and 95% confidence interval.

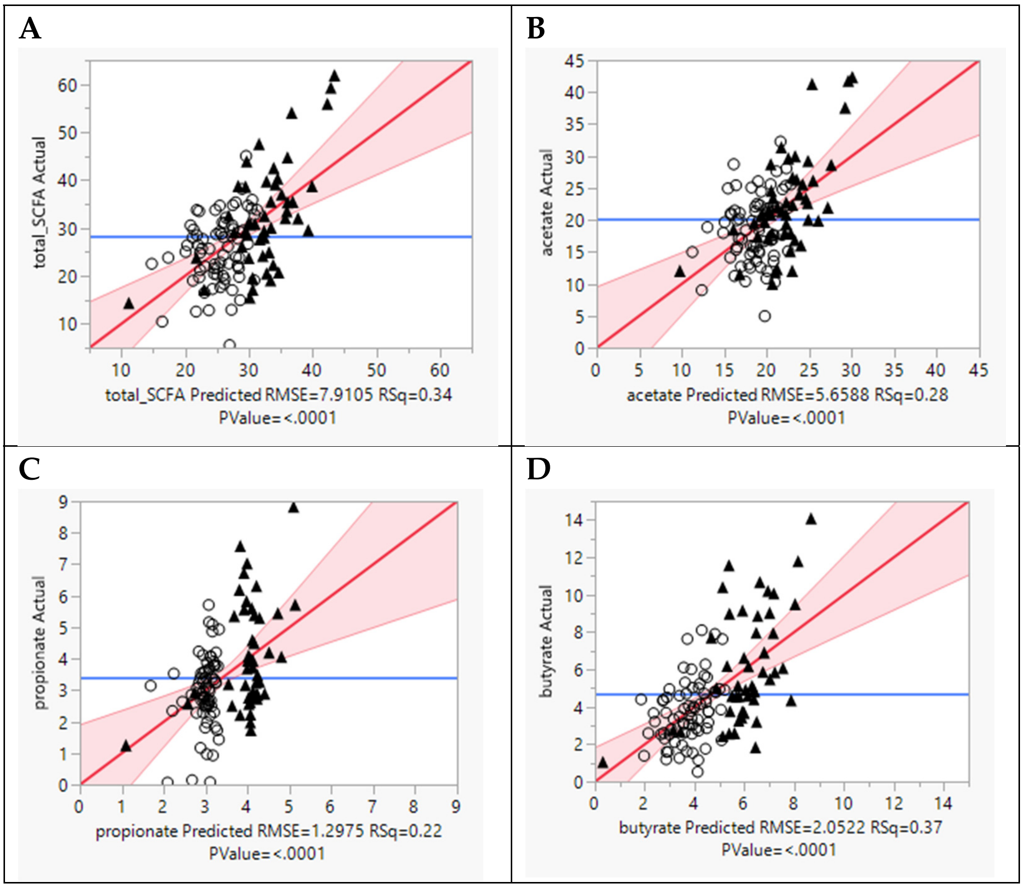

Figure S2. Multiple linear regression models of total and individual SCFAs using sex, and microbial clusters 15, 13 and 2. A) Total SCFA. B) Acetate. C) Propionate. D) Butyrate. Open circles – women; closed triangles = men. All models are significant at $p < 0.0001$.

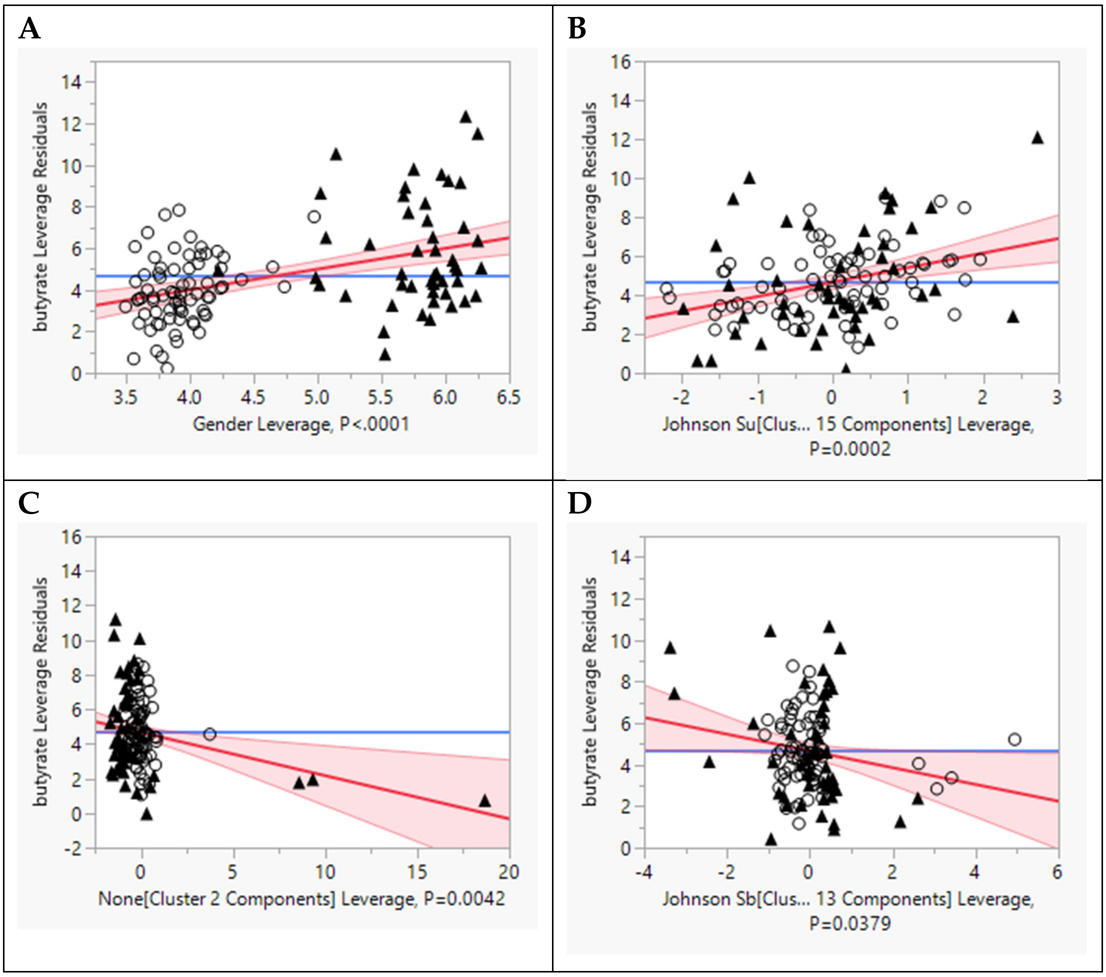

Figure S3. Multiple linear regression models of butyrate using sex, and microbial clusters 15, 13 and 2. A) Sex. B) Cluster 15. C) Cluster 2. D) Cluster 13. Open circles – women; closed triangles = men. All models are significant at p <0.0001.

Table S1. Dietary intake averaged from three 24-hour recalls obtained using ASA-24 during each intervention period

		Resistant Starch (RS) n=33	Control n=33
**Macronutrients**			
Energy	kcal/d	2659.1 ± 836.6	2580.2 ± 642.5
Carbohydrate	g/d	341.0 ± 113.3	338.1 ± 90.2
Protein	g/d	115.7 ± 34.1	112.4 ± 34.7
Total fat	g/d	95.7 ± 41.0	88.9 ± 26.8
Dietary fiber	g/d	56.4 ± 19.4	29.6 ± 15.2
	g/1000 kcal	21.4 ± 3.2	11.2 ± 3.6
**Food type contributing dietary fiber**			
Fruit	cup-equiv/d	1.9 ± 1.9	1.4 ± 1.2
Vegetables	cup-equiv/d	2.1 ± 1.1	1.9 ± 1.1
Legumes	cup-equiv/d	0.5 ± 0.6	0.5 ± 0.7
Nuts & seeds	oz-equiv/d	1.4 ± 2.2	2.5 ± 3.8
Whole grains	oz-equiv/d	0.9 ± 1.2	1.2 ± 1.4
Refined grains	oz-equiv/d	7.1 ± 2.8	7.7 ± 3.2

Footnote: Values are mean ± standard deviation

Table S2. DESeq2 results of effect of treatment on microbial taxa

Direction Change	log2FoldChange	pvalue	Padj	Taxon
RS vs. C				
Direction Change	log2FoldChange	pvalue	Padj	Taxon
Increase	0.661	**0.003**	0.07	g__*Faecalibacterium*
Increase	1.352	**0.001**	**0.031**	g__*Ruminococcus*
Increase	1.3	**<0.001**	**0.022**	g__*Gemmiger*
Increase	1.001	**0.046**	0.742	g__*Bifidobacterium*
Decrease	-1.436	**0.018**	0.37	g__[*Ruminococcus*]†
RS vs. Pre-RS				
Direction Change	log2FoldChange	pvalue	Padj	Taxon
Increase	0.986	**<0.001**	**<0.001**	g__*Faecalibacterium*
Increase	1.923	**<0.001**	**<0.001**	g__*Ruminococcus*
Increase	1.517	**0.002**	**0.03**	g__*Roseburia*
Increase	1.619	**<0.001**	**<0.001**	g__*Gemmiger*
Increase	2.839	**<0.001**	**<0.001**	g__*Bifidobacterium*
Increase	1.166	**0.011**	0.152	f__*Rikenellaceae*
C vs. Pre-C				
Direction Change	log2FoldChange	pvalue	Padj	Taxon
Increase	0.614	**0.028**	1	g__*Bacteroides*
Increase	1.756	**0.001**	**0.048**	g__*Bifidobacterium*

† g__[*Ruminococcus*] belongs to family *Lachnospiraceae*. Abbreviations: Control (C), Resistant Starch (RS).

Table S3. Short-chain fatty acid concentrations

Treatment	Total SCFAs (pmol/mg)	Acetate (pmol/mg)	Butyrate (pmol/mg)	Propionate (pmol/mg)	Acetate (%)	Butyrate (%)	Propionate (%)
Pre-C	27.9 ± 7.3	20.6 ± 5.3	4.1 ± 1.9	3.2 ± 1	73.9 ± 6	14.5 ± 4.3	11.5 ± 2.4
Control	28.9 ± 9.6	20.5 ± 6.5	4.7 ± 2.7	3.7 ± 1.4	71.5 ± 6.4	15.8 ± 4.5	12.8 ± 3
Pre-RS	27.1 ± 11	19.2 ± 7.1	4.8 ± 3	3.1 ± 1.7	72.7 ± 8.5	16.4 ± 5.8	10.9 ± 4.6
RS	29 ± 10.4	20.4 ± 7.3	5 ± 2.5	3.5 ± 1.6	70.8 ± 5.6	16.9 ± 4.5	12.4 ± 3.2

Footnote: Values shown are mean ± standard deviation. Abbreviations: Before Control (Pre-C), Before Resistant Starch (Pre-RS), Resistant Starch (RS), short-chain fatty acids (SCFAs).

Table S4. Variable Clustering of Microbial Taxa

Cluster	Members	RSquare with Own Cluster	RSquare with Next Closest	1-RSquare Ratio
1	f__Christensenellaceae_203f3186ffa10b8d5f79bb46f6131932_per	0.9	0.08	0.11
1	g__Leuconostoc_4c28ca21cfe8aae96cb4fdfda962e00f_per	0.83	0.06	0.19
1	g__Anaerotruncus_b0380be317ab3d535fda80361fef72dc_per	0.82	0.06	0.19
1	g__Methanobrevibacter_e10723224eb30a57e03cfb25428192cf_per	0.71	0.03	0.29
1	f__Mogibacteriaceae_0acda530a021f0442db672257bd840f0_per	0.65	0.16	0.42
1	g__Oscillospira_c9b3d3582dd7a6714e0f50fc0aae5358_per	0.53	0.12	0.54
1	g__Bilophila_8844ba6ff4d7fd8255aca7553a70aa8a_per	0.36	0.1	0.71
1	g__Dorea_da2938f07b3eb5e35cffd90b0d8f4afe_per	0.3	0.07	0.75
1	g__Streptococcus_0718e6f569c5f42fe6114ed5288343b8_per	0.41	0.23	0.77
2	f__Victivallaceae_a91b2451ac106c648fb81c8e49d6e0d6_per	0.95	0.05	0.05
2	c__Alphaproteobacteria_7751a67b387f17023737d1585e35db46_per	0.9	0.06	0.11
2	f__Anaeroplasmataceae_c989da24af206e59a99e928bccbdcc55_per	0.78	0.05	0.23
2	o__Burkholderiales_5b2186549c92b9ec4d264e623f029d51_per	0.77	0.04	0.24
2	k__Bacteria_02c4a0e511d46189db78ebc317e63b6b_per	0.73	0.07	0.29
2	g__Desulfovibrio_3baebc874e88c0cb86678ea3ab710b51_per	0.71	0.1	0.33
2	o__ML615J28_8d1e851ccf8a60ba51dd032cf3e71106_per	0.31	0.11	0.77
2	o__Bacteroidales_d437a6d10db675a31fb9592048042dd4_per	0.02	0.01	0.99
3	f__Erysipelotrichaceae_1a44da1de60bb0a2f54b6bb01e03544f_per	0.64	0.06	0.39
3	g__Odoribacter_fd90fd536d69fbb927700a4f816b5437_per	0.5	0.1	0.55
3	f__Barnesiellaceae_a5144553aa7bd505c53b2005926ac4bf_per	0.42	0.15	0.68
3	g__Sutterella_f906f7c32eca8e6d5b140f23676d4cb2_per	0.38	0.1	0.68
3	o__RF39_223de6c418e8ae64c4e8d5ca99fe21ea_per	0.27	0.14	0.85
3	g__Clostridium_3c36049d1d75e84b7fea2e92b2c38831_per	0.17	0.04	0.86
4	f__Peptostreptococcaceae_f6bc3b448024a35619eff2bb01a03279_per	0.99	0.07	0.01
4	g__Lachnobacterium_2b91198581ae3c6f6ecff1dd239de5ac_per	0.99	0.07	0.01
4	g__Megasphaera_2cb1b23a0065cd57f3c80c2098e519fc_per	0.97	0.15	0.03
4	f__Clostridiaceae_8a02d54c269bac1e3d87b352b795489e_per	0.03	0.01	0.98
5	g__Eggerthella_8ab2345734740ca1475a36fdc37c6209_per	0.82	0.02	0.18
5	g__Collinsella_cf19aa76c5793bbcbdfe8b6e4e497245_per	0.74	0.02	0.26
5	f__Fusobacteriaceae_4865f293826e093f78ce53f60e06604c_per	0.62	0.03	0.39
5	g__Paraprevotella_622cff538fea6cf8fece6dc45b54a836_per	0.39	0.02	0.63
6	g__Clostridium_efc3d396dde8734eb0d089c04450d367_per	0.59	0.04	0.42
6	g__Veillonella_4226524157c73e083234aea083217bab_per	0.4	0.02	0.61
6	g__Faecalibacterium_5c4e76bbeb28b2680f454895ec903c48_per	0.46	0.12	0.62
7	g__human_3e40c62f5d6da776aaf8060a9403d763_per	0.84	0.07	0.17
7	g__Acidaminococcus_57629fd74969b126a829f2514e883c63_per	0.74	0.1	0.29
7	f__Coriobacteriaceae_8bfce0da546a02a357896cdb978cec3d_per	0.33	0.03	0.69
7	g__Ruminococcus_7171cdaa3c82bba37a16f57143794b42_per	0.25	0.09	0.83
8	o__Clostridiales_8c42ed4e97c974ba144a2bb088a5fb70_per	0.66	0.15	0.41

8	g__Bacteroides_f128b8cdf56dad0ff08cf868e6663af7_per	0.59	0.12	0.47
8	f__Ruminococcaceae_df89fce167303ab357d1ff2779dc6198_per	0.63	0.22	0.47
8	f__Rikenellaceae_e399cfd28cb3218d9c310ca108f8704a_per	0.29	0.05	0.75
8	g__Butyrivibrio_aa16e4a118d63f745fe047b885e0272e_per	0.33	0.12	0.76
9	g__Anaerofustis_a792724b1ca76b19670d137432e8b69e_per	0.86	0	0.14
9	g__SMB53_dde86150efe323dbe1986fdc1a3aaf7b_per	0.82	0.03	0.18
9	g__Lachnospira_17dba817f4132be19c8d41450fdbd7cc_per	0.2	0.04	0.84
10	g__Barnesiella_1c6db45aef5a29ae4089ca4f3bf64720_per	0.68	0.09	0.35
10	o__RF32_4acc4d01f5d8d1edbf2a251c434386d4_per	0.61	0.12	0.45
10	g__Anaerostipes_aec581a4afb8a54d6939718f1f9fcc71_per	0.15	0.03	0.88
11	g__Blautia_b35811152097574225863af5ed2ccc7c_per	0.61	0.09	0.43
11	g__Clostridium_720a468b4b430d4ab966842d8ed9cfc5_per	0.55	0.01	0.46
11	g__Akkermansia_129a6f55c08bef50c705ae8f67515c41_per	0.54	0.03	0.48
12	g__Turicibacter_906d01920ac6d2294524968f3bda9e08_per	0.71	0.13	0.33
12	g__Lactococcus_d526eeb78ad98f4a50742189333e4f6a_per	0.65	0.08	0.38
12	g__Gemmiger_3928a4255d8b9bed26ef0020f58f4220_per	0.23	0.07	0.83
13	g__Butyricicoccus_e0812126349e0f12a7e57b76ce570e35_per	0.66	0.05	0.36
13	g__Coprobacillus_f8817a191dcc7efe5f482e30e232bc4e_per	0.62	0	0.38
13	f__Lachnospiraceae_1f9c02db43c1e5681fe0e9f7e4fad5ea_per	0.09	0.03	0.94
14	g__Dialister_fecea4659c5bf13af5c0a1b3aedc543b_per	0.6	0.06	0.43
14	g__Ruminococcus_6940da4d95e8cdadfadc5c7e1b7f87cf_per	0.47	0.04	0.56
14	g__Fusobacterium_e49627cc0a046ee60bcc135d44b6d3d8_per	0.4	0.09	0.66
15	g__Roseburia_cb4ccead11fd04cab24fe711fd909f27_per	0.53	0.06	0.5
15	g__Ruminococcus_311d9d226a66ab84f1a8433cede7736c_per	0.43	0.16	0.68
15	g__Parabacteroides_f76b8710c3b2198831504bd01ffae4ed_per	0.37	0.13	0.73
15	g__Butyricimonas_1900db1f5b39fd3ce2e088d66e0c05ab_per	0.3	0.06	0.74
15	g__Lactobacillus_a59d195eebab2efb90ff4a0addba5771_per	0.13	0	0.88
16	f__Gemellaceae_224a108589d9e4b62a8b3fcef3f0a9da_per	0.64	0.02	0.37
16	g__Eubacterium_320423dc5590a7d4a98086920cebd972_per	0.64	0.07	0.39
17	f__Paraprevotellaceae_3620984cfb2002b931b33b6690de016c_per	0.79	0.07	0.23
17	f__S247_df90fc79bc38a50c40533a043e7cb46b_per	0.75	0.24	0.33
17	g__Phascolarctobacterium_0ce3ba441b32bcf0771cde152e2b5653_per	0.27	0.09	0.81
18	g__Prevotella_0264c34983e970203513d396a0a48a3c_per	0.68	0.09	0.36
18	g__Prevotella_5be9b95af0add8f78178c24766ec4a2b_per	0.63	0.13	0.42
18	g__Bifidobacterium_34207721d3d8489c257c3d33920b0eb1_per	0.17	0.02	0.84
19	g__Slackia_c82e12406ddc70448f2302ea91056172_per	0.61	0.01	0.39
19	g__Alistipes_2726333037520ac21b775f84caa83edf_per	0.61	0.07	0.41
20	p__Firmicutes_423cb807aa790bc1a08a54db03dd3437_per	0.7	0.01	0.3
20	o__YS2_591192c6fff8932e640709000f76a462_per	0.7	0.04	0.31
21	g__Oxalobacter_d1452c3438020ebe1396c8ee1b01b5d4_per	0.52	0	0.48
21	g__Coprococcus_8c5e689e16544c2b0fd718250c392c06_per	0.52	0.07	0.51

Footnote: Microbial taxa names shown as: taxonomic level (g: genus, f: family, o: order, c: class, p: phylum)_taxonomic name_sequence variant (SV) number_percent (relative abundance). g__Ruminococcus_6940da4d95e8cdadfadc5c7e1b7f87cf_per belongs to family *Lachnospiraceae* and g__Ruminococcus_7171cdaa3c82bba37a16f57143794b42_per belongs to family *Ruminococcaceae*.
